# Primary Tumors of the Spleen

**Published:** 2009-06

**Authors:** C. Fotiadis, I. Georgopoulos, C. Stoidis, P. Patapis

**Affiliations:** *3rd Department of Surgery, National and Kapodistrian University of Athens, Medical School, "Attikon" University Hospital of Athens, Greece*

**Keywords:** spleen, tumors, primary, metastatic

## Abstract

Tumors of the spleen are rare compared to the incidence of such tumors in other parenchymatous organs. Their classification has varied with both time and author. They can be divided into two main categories: nonlymphoid and lymphoid. The most common nonlymphoid tumors are the vascular tumors which include benign and malignant haemangiomas, littoral cell angiomas, lymphangiomas and haemangioendotheliomas. The remaining nonlymphoid tumors, such as fibrosarcoma, neurinoma, and lipoma are very uncommon. The lymphoid tumors include Hodgkin’s and non Hodgkin’s lymphoma, histiocytic lymphoma and plasmacytoma. Metastatic tumors to the spleen mainly originate from melanoma, breast and lung lesions. However, metastases to the spleen are rare compared to those of other parenchymatous organs.

## INTRODUCTION

Primary splenic neoplasms are uncommonly encountered in the practice of general surgical pathology, but when present they may require splenectomy for either diagnosis or treatment. The fragile spleen, hidden in its left upper quadrant recess, always presents an interesting and difficult challenge to the surgeon, while the presence of a neoplasm makes it an even harder challenge.

## CLINICAL FEATURES-DIAGNOSTIC TESTS

No constant clinical features can be fitted to the entire group of splenic tumors. Splenomegaly is the commonest of findings. Upper quadrant discomfort, pain or tenderness may also be present. Anaemia, granulocytopenia and thrombocytopenia are also possible, depending on the localization and the size of the lesion. In case of malignancy, splenomegaly may be accompanied by signs of systemic involvement such as fever, cachexia and pleural infusion. Massive splenomegaly (over 3000 gr) may cause adjacent viscera displacement and pressure, leading to a variety of symptoms, including dyspnoea, shoulder pain and constipation. The most useful imaging test available today is the computed tomography (CT) scan. Histological examination following surgical removal, seems to be the most accurate technique to establish diagnosis.

## PRIMARY TUMORS OF THE SPLEEN

### Vascular tumors

Benign

- Haemangioma
- Lymphangioma
- Littoral cell angioma
- Haemangioendothelioma

Malignant

- Haemangiosarcoma
- Littoral cell angiosarcoma

### Lymphoid tumors

- Hodgkin’s disease
- Non-Hodgkin’s lymphoma
- Plasmacytoma
- Castleman’s tumor
- Localized reactive lymphoid hyperplasia
- Inflammatory pseudotumor

### Non-lymphoid tumors

- Lipoma, angiolipoma
- Malignant fibrous histiocytoma
- Fibrosarcoma
- Leiomyosarcoma
- Malignant teratoma
- Kaposi’s sarcoma

## BENIGN VASCULAR TUMORS OF THE SPLEEN

### Haemangioma

**Figure 1:**
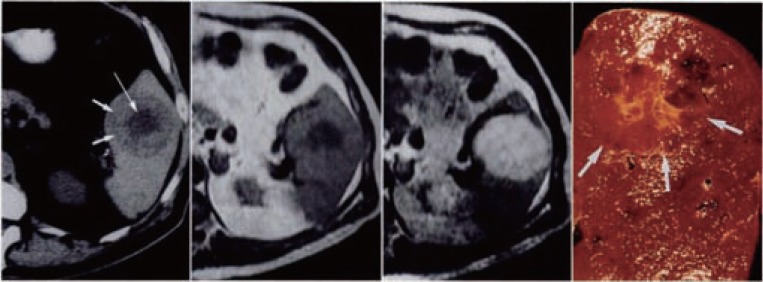
Splenic haemangioma. Unenhanced CT scan shows a round splenic lesion with lower attenuation in the centre (arrow) than in the periphery. Weighted MR image shows that centre has low signal intensity but periphery is nearly isointense in the spleen. Lesion becomes bright on T2 weighted MR image. Cut surface of gross specimen shows an unencapsulated hemorrhagic lesion (arrows) with central stellate scar. Source: D.Disler, F.Chew, Radiologic-Pathologic Conferences of the Massachusetts General Hospital.

Splenic haemangiomas (Figure 1) remain the most common benign neoplasms of the spleen, although these are infrequent lesions, with larger autopsy series reporting the incidence at 0.02% to 0.16% (1). In the vast majority of cases (2-4) they are incidental surgical, radiological, or autopsy findings. Symptoms or physical signs directly related to the presence of a splenic haemangioma are very uncommon. In the larger series (2), of 32 patients only 6 presented with abdominal symptoms and of these patients only 4 had a palpable spleen. Clinical recognition occurs most commonly in middle-aged adults. There is also a significant variability in the age presentation of this entity, with an average of detection/presentation of between 51 to 63 years of age (2, 3). A gender or race predilection has not been reported. Splenic haemangiomas appear on CT scans as single or multiple lesions that are usually homogenous, hypodense or multicystic. They may contain calcifications and generally demonstrate peripheral enhancement after intravenous contrast injection. Ultrasound often demonstrates round, echogenic masses, with or without cystic areas. They consist an intermediate form, between true tumors and dysplasias, which occasionally resemble pseudotumors. Even if they are usually labelled tumors, they are in fact dysplasias with excessive vascular production. Haemangiomas are most often visible macroscopically as blood-filled cysts, occurring either singly or in groups. As may be expected, the size of these lesions varies greatly between those removed because of symptoms and those found incidentally at surgery or autopsy. Histologically, they may be capillary-type or cavernous in pattern. The usual picture is that of vascular spaces lined by a single layer of bland endothelial cells, without mitoses. Thrombosis and infraction occur in many cases, presumably because of abnormalities in the vascular supply of the tumor. When completely organized, infracted haemangiomas may resemble a fibroma or a healed, simple infract of the spleen. The cells of normal red pulp sinusoids are unique to the spleen, showing biphenotypic immunoreactivity for vascular and histiocytic markers (5). Study of this immunophenotypic pattern raises the possibility that splenic haemangioma may derive from a combination of splenic venous structures, as well as splenic sinusoidal cells. It can also contribute to differentiating the lining cells in haemangiomas from the endothelial cells present in littoral cell angiomas and splenic hamartomas. Hamartomas are also distinguished from hemangiomas by their usually more poorly defined margins. Although benign, splenic haemangioma is not entirely without complications as larger lesions may rupture with resulting intra-abdominal haemorrhage. The potential for malignant transformation of splenic haemangioma to angiosarcoma is unknown. Malignant transformation is stated to occur more frequently with a large splenic Haemangioma or when the spleen is diffusely involved. Their controversial malignant potential, along with the danger of spontaneous rupture, makes splenectomy the recommended treatment for all cases of splenic haemangioma.

Diffuse haemangiomatosis of the spleen is a very rare variant of splenic haemangioma in which the spleen is diffusely replaced by blood vessels of varying caliber (6). Typically, this entity is associated with generalized angiomatosis of other hematopoietic organs such as the bone marrow and the liver (7). In addition, hematologic abnormalities, including anaemia, thrombocytopenia and severe coagulopathies, such as microangiopathic haemolytic anaemia, frequently accompany these lesions.

### Lymphangioma

Lymphangioma of the spleen is even less common than haemangioma of the liver, with clinical manifestations ranging from insignificant incidental findings to large, symptomatic multicystic splenic masses requiring surgical intervention (8-10). The latter are seen in conjunction with a childhood syndrome, disseminated lymphangiomatosis, in which the lymphangiomatous process diffusely involves other sites or organs, such as the bone, soft tissue, or viscera (11). Splenic lymphangiomas may be single or multiple. Usually they are small multicystic, subcapsular proliferations that are difficult to distinguish from either haemangioma or mesothelial cysts. Unlike splenic haemangiomas, which typically show random localization in the spleen, splenic lymphangiomas are frequently subcapsular or trabecular in location, regions where splenic lymphatics are normally situated. In the past many mesothelial cyst where mistaken for lymphangiomas (12), until careful immunophenotypic analysis of these lesions showed evidence of mesothelial rather than lymphatic origin the cystic lining cells. Histologically, the neoplasm consists of thin-walled cystic structures of varying size lined by flat endothelium, filled with a pink eosinophilic proteinacious material, devoid of red blood cells. With careful inspection, foci of plump lining cells, often forming striking papillary projections, are typically seen. Caution should be exercised in distinguishing these papillary areas from angiosarcoma. Definitive distinction between lymphangiosarcoma and angiosarcoma is currently not possible; therefore cases such as these are best classified as angiosarcoma.

### Littoral Cell Angioma

Littoral cell angioma (LCA) of the spleen is a vascular tumor that represents a lesion unique to the spleen, without a soft tissue counterpart (13). LCAs are believed to derive from the lining cells of the splenic red pulp sinuses, which normally demonstrate both endothelial, and histiocyte/macrophage appearances and properties. These lesions can occur in all age groups without sex predilection, but most commonly present in middle age. They are typically identified in patients undergoing splenectomy for splenomegaly of unknown origin or radiographically during workup for other abdominal processes (14). They can be associated with splenomegaly leading to thrombocytopenia or anaemia. Cases of patients presenting fever that resolve after splenectomy have been reported. Interestingly, 9 of 29 well-documented cases of LCA reported in the literature are described as being associated with other cancers (15). No specific genetic or molecular abnormalities are found, to suggest a reason for this association with malignancy. It may be that these LCAs where simply identified by chance in this subset of patients undergoing extensive radiologic investigation for other disease. However, some authors recommend close follow-up and careful investigation in search of a second visceral neoplasm, when an LCA is identified. Macroscopically, splenic involvement it characterized by multiple, spongy, cystic blood filled, circumscribed nodules, ranging in size from 0.2 to 9 cm. Less commonly, these lesions can be solitary or completely replace the splenic parenchyma. Microscopically, these lesions correspond to a complex anastomosing network of vascular spaces of varying size, some with narrow compressed lumens; others are composed predominantly of large dilated blood-filled spaces. A very characteristic finding of the LCAs is focal aggregates of eosinophilic globules 0.5 to 2 μ in size, which often entirely fill the tumor cell cytoplasm (16). The globules most probably originate from the phagocytised red blood cells, lymphocytes and plasma cells. Several morphologic features of LCA, including the papillary projections and occasional solid areas, can make differentiation from angiosarcoma sometimes challenging. Careful analyses of the morphologic features of the lesion, as well as immunohistochemical analysis are of diagnostic utility in distinguishing these entities.

## MALIGNANT VASCULAR TUMORS OF THE SPLEEN

### Angiosarcoma

**Figure 2:**
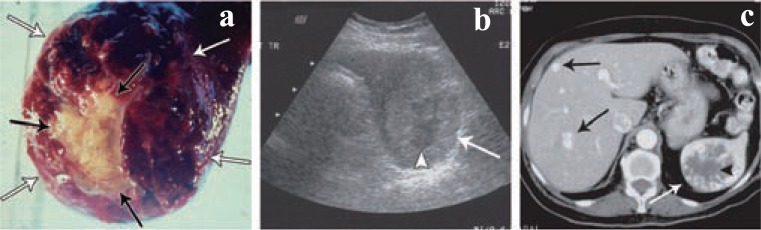
Angiosarcoma of the spleen. Source: W.M.Thomson, Angiosarcoma of the spleen: Imaging characteristics in 12 patients. Radiology. 2005; 235:106-115.

Primary angiosarcoma (Figure 2) of the spleen is a very rare and aggressive neoplasm with a high metastatic rate and dismal prognosis. The median age of patients with splenic angiosarcoma is typically 50-60 years (17, 18). While there is no apparent gender predilection, it has been found that women present at a statistically significant older age than men. Recently, there has been emphasis on the association of the splenic angiosarcoma with environmental or workrelated factors such as thorium dioxide or monomer vinyl chloride. Some small series have also noted histories of exposure to ionizing radiation prior to the development of splenic angiosarcoma (19). No clear relationship between prior chemotherapy has been established to date. More often the spleen is affected when angiosarcoma develops in another organ, usually the liver. Angiosarcoma is typically associated with significant symptomatology. The majority of patients experience abdominal pain, typically localized to the left upper quadrant, with some reporting weakness or fatigue, weight loss and fever. Most patients have splenomegaly and spontaneous splenic rupture with haemoperitoneum is commonly seen. Spleens involved in angiosarcoma reveal 85% to 90% of cases with splenic weights well over 250 g (normal weight 80-150 g). Spleens that rupture are on average much larger than those that do not. Abnormal laboratory findings are seen in nearly all patients, with a normochromic, normocytic anaemia being most common, followed by thrombocytopenia. White blood cell counts are generally depressed and patients present with pancytopenia; however, examples of elevated white cells counts or even thrombocytosis have been reported. Some not that common laboratory findings are coagulopathy (elevated PT/PTT) or microangiopathic hemolytic anemia. Histologically angiosarcoma of the spleen has an extremely varied appearance, not only seen from case to case, but also within a given tumor, making diagnosis based on light microscopy alone difficult. Nearly all cases demonstrate a spongiform or honeycomb-like proliferation comprised of an irregularly anastomosing network of slit or capillary-like spaces. These patterns are similar to those seen in other benign splenic vascular neoplasms; what distinguishes these cases as angiosarcoma is the cytological atypia of the lining cells. In contrast to them, mitotic figures and multinucleated, bizarre, tumor giant cells are frequently seen. While some regions can be easily recognized as malignant, the lack of a vasoformative pattern may make identification as angiosarcoma difficult. Immunohistochemical studies are often useful in this regard, confirming the vascular nature of these tumors Prognosis is dismal with the majority of patients rapidly dying of disseminated disease. Most studies reveal that the majority of patients have median survivals of around 5 to 6 months with nearly all patients dying within 3 years. Treatment, which in most cases involves splenectomy followed by radiation and/or chemotherapy, does not affect these numbers. Metastasis is early and frequent in angiosarcoma, with rates of metastasic spread reported from 70% to 85%. Common sites of metastasic disease are the liver, lung, lymph node and bone; less frequent sites are the brain, soft tissue and adrenal gland. Surprisingly, metastasic disease at diagnosis does not appear to be associated with a higher death rate or accelerated demise. Alternatively, the profound haematological abnormalities accompanying the tumor may play a significant role in the patients’ ultimate demise. The only reasonably consistent factor in favour of a somewhat better prognosis is tumor size less than 5 cm.

### Littoral Cell Angiosarcoma

Rarely malignant splenic vascular tumors are encountered with the morphological features of littoral cell angioma, yet which display malignant nuclear cytology and an infiltrative or solid growth pattern (21). Macroscopically, these lesions show an ill-defined, non-lobulated pattern with alternating dark red-spongy nodules and interspersed solid white areas. Light microscopic examination reveals a diffuse growth pattern, with vascular spaces of varying space and size lined by bland, grooved lining cells resembling those of typical littoral cell angioma. In at least one case, clinical behaviour intermediate to that of LCA or angiosarcoma was identified. This lesion was not rapidly fatal and recurred 8 years after splenectomy, with an abdominal mass and multiple liver metastases, resulting in the patient’s death. These tumors have been regarded as malignant counterparts of LCA. However, studies showing a splenic sinus lining cell origin of many primary angiosarcomas make reliable or meaningful distinction between these entities difficult. Their entire immunohistochemical profile has been reported to be consistent with that of a classic littoral cell angioma. Lesions like these may represent a biologically distinct entity from angiosarcoma. However, since biological behavior cannot be predicted entirely based on biological features, long term follow-up for these patients, especially for those with atypical histology, is recommended.

### Haemangioendothelioma

Haemangioendothelioma of the spleen is a controversial entity that may represent a vascular lesion with morphologic and clinical properties that are intermediate between those of the haemangioma and angiosarcoma (22, 23). They typically demonstrate a range of microscopic features with ill-defined vascular spaces, epithelioid morphology with mild cellular atypia, a low mitotic rate, and absence of necrosis. Their malignant potential is best described as borderline or intermediate. Their clinical findings include splenomegaly or a palpable mass. Chronic anaemia has been reported in a few cases, while there is a case report of a splenic rupture. Although most authors feel that the majority of such lesions most likely represent examples of angiosarcoma, other variant of haemangioendothelioma, particularly those with spindle and epithelioid morphology or an endovascular papillary agioendothelioma pattern probably do represent distinct entities that warrant separation from angiosarcoma, as with comparable lesions in soft tissue. With this in mind, only rare reports of such splenic lesions exist in the literature. Several well-circumscribed haemangioendotheliomas with spindle and/or epithelioid morphology and no evidence of metastatic spread have been described, while other cases with similar histological features have shown more aggressive behaviour, typified by extra-splenic spread. Aside from conventional morphologic assessment, reliable indicators for a malignant phenotype or aggressive biological potential do not exist. Careful attention to morphologic features and correlation with clinical findings is necessary. Until better markers to predict clinical behaviour exist, the presence of atypical cytological features justifies, at the least, close monitoring of such patients.

## LYMPHOID SPLENIC NEOPLASMS

### Hodgkin’s disease

The spleen is often the site of secondary involvement by Hodgkin’s disease (23). Overall, lymphoid malignancies are uncommon as primary splenic tumors. Primary splenic involvement incidence has been studied in our series of splenectomies, showing that in 59% of the cases the spleen was intact, while in 41% was neoplasmatically active. It has always been difficult to evaluate the presence or absence of the disease in the spleen, using non-invasive methods. Most published series show disappointing results in the assessment of the histological state of the spleen using CT. Histological examination following surgical removal, seems to be the most accurate technique to establish splenic involvement. When lymphoma does involve the spleen, either as a primary or secondary process, the white pulp is involved first. There may be diffuse involvement or there may be large, irregular tumor masses. This will in most cases lead to an excessive splenomegaly. A large spleen is not always a malignant one. In our series of histologically examined healthy spleens, 30% had increased weight (200-300 g), while 10% presented with excessive splenomegaly (300-800 g). On the other hand, normal spleen size does not exclude malignancy. Splenic involvement of is associated with increased incidence of liver and bone marrow involvement.

### Non-Hodgkin’s Lymphoma

Splenic involvement is reported in 50-80% in patients with non-Hodgkin’s lymphoma. Primary splenic involvement is much less common. Usually a large spleen with small lesions, possibly microscopic, in other organs, raises the suspicion for a primary splenic origin. As in Hodgkin’s lymphoma, size and weight of the spleen may vary, while they do not correlate with the prognosis of the disease. Macroscopically there can be a diffuse involvement, small nodular lesions, large nodular lesions or a large tumor mass. Many infracts or blue subcapsular dots can be seen. The malignancy of these lesions can be rated following the Kiel pattern or the IWF (International Working Formulation) pattern. Benign lymphoid lesions may also cause splenomegaly and may mimic lymphoma. Reactive lymphoid hyperplasia and inflammatory pseudotumor have been briefly described. Angiofollicular lymphoid hyperplasia, also known as Castleman’s tumor may also affect the spleen, either as a solitary lesion or as a part of a diffuse lymphoid hyperplasia syndrome. Castleman’s tumor is thought to be a true lymphoid hamartoma, but does not resemble the follicular pattern of splenic hamartoma described by Berge. Primary plasmacytoma of the spleen is exceedingly rare and is not macroscopically recognizable, but is easily identified histologically as a plasma cell neoplasm. The spleen may be involved as a primary, isolated site or as a part of generalized myelomatosis.

### Non-lymphoid tumors

Lipoma of the spleen is a rare entity. Usually it appears as round mass of lipoid tissue. Angiolipomas have occasionally been reported as well. A number of cases of primary splenic malignant fibrous histiocytoma have also been described. This distinct, polymorphic sarcoma usually occurs in the soft tissues, but has also been found in a number of organs, including the spleen. Massive splenomegaly is usual, while these tumors tend to be very aggressive. Malignant fibrous histiocytoma may be misdiagnosed as fibrosarcoma or leiomyosarcoma, both of which occur in the spleen, but have not been well documented. Neurinoma or schwannoma of the spleen has also been described. There is a single report of a malignant teratoma, with papillary carcinoma, spindle cell carcinoma and cartilaginous tissue.

Recently, incidence of Kaposi’s sarcoma has greatly increased, mainly in association with human immunodeficiency virus infection (HIV) and the acquired immunodeficiency syndrome (AIDS). It is a spindle cell malignant neoplasm, characterized by vascular spaces not generally lined by endothelial cells, which may contain blood. Kaposi’s sarcoma has not been described as a primary splenic tumor, but is seen in patients in whom the spleen is involved as part of a general sarcomatosis.

Hamartomas or Splenomas of the spleen are rare, benign splenic lesions. They are not neoplasms, but dysplastic nodes that may be misdiagnosed as neoplasms. Most of the cases have been found as autopsy material. Their incidence has been estimated at 3 in 200.000 splenectomies. They occur equally in males and females, at all ages, but particularly in older people. Typically, they appear as well-circumscribed lesion, which appears darker than the surrounding spleen. Their size varies from a few mms to many cms. They are easily recognized microscopically, because of the distinct border and the slit like and tortuous endothelial-lined spaces. Sometimes they can cause splenomegaly, but hypersplenism is rare. Cases of rupture of a solitary splenic hamartoma, with life-threatening haemoperitoneum, have been reported in the literature.

## METASTATIC TUMORS INVOLVING THE SPLEEN

Splenic metastases are very rare. Berge reported the overall incidence of splenic involvement as 7.1% in 7165 autopsy cases with various cancers and the incidence of splenic metastasis from colon and rectal carcinomas as 4.4% and 1.6% respectively. However, he did not report any case of solitary splenic metastasis. Anatomical, histological and functional features of the spleen have been speculated as the reason of the rarity for solitary cancer metastasis. Particularly frequent carcinomas of origin are those of breast, lung and melanomas. Direct extension from retroperitoneal tumors and pancreatic carcinoma may be found. Virtually all primary tumors have been shown to be able to metastasize to the spleen, while in most cases splenic metastases occur as a component of disseminated disease. Most cases are reported as asymptomatic and the diagnosis is usually made by the imaging studies such as abdominal ultrasound (US) or computerized tomography (CT) during the evaluation of a rising CEA level in the postoperative follow-up period of colorectal cancer patients. However, splenomegaly, left pleural effusion, spontaneous rupture has been reported.

## DISCUSSION

Neoplasms of the spleen are rare entities, which may be a diagnostic challenge. Most of them are asymptomatic and are found incidentally after splenectomies. However, the fragile spleen as well as the malignant potency of a few splenic neoplasms makes their fast and accurate diagnosis imperative for more than a few patients.
